# The Formation of Nanoparticles and Their Competitive Interaction with Twins during Eutectic Si Growth

**DOI:** 10.3390/ma11081404

**Published:** 2018-08-10

**Authors:** Wei Wang, Fengxiang Guo, Zhigang Gai, Tao Zhang, Jianguo Tang, Xuelei Tian, Wenqing Liu

**Affiliations:** 1 College of Materials Science and Engineering, the National Base of International Science and Technology Cooperation on Hybrid Materials, Qingdao University, 308, Ningxia Road, Qingdao 266071, China; tang@qdu.edu.cn; 2 Institute of Oceanographic Instrumentation, Qilu University of Technology (Shandong Academy of Sciences), Shandong Provincial Key Laboratory of Marine Monitoring Instrument Equipment Technology, National Engineering and Technological Research Center of Marine Monitoring Equipment, Qingdao 266001, China; zhiganggai@126.com (Z.G.); zt@sdioi.com (T.Z.); 3 College of Materials Science and Engineering, Shandong University, 17923, Jingshi Road, Jinan 250061, China; 4 Key Laboratory for Microstructures, Shanghai University, Shanghai 200444, China; wqliu@staff.shu.edu.cn

**Keywords:** nanoparticles, twins, TEM, atom probe tomography

## Abstract

In order to investigate the competitive interaction between nanoparticles and twin, the eutectic Si microstructures in Al–10Si (wt. %) base alloys with exclusive and combined addition of Sr and Sb are characterized by combined TEM and atom probe tomography (APT). The chemical short range order in Sb–Sb and Sb–Sr pairs is revealed by ab initio molecular dynamics simulation, which promotes the formation of clusters and nanoparticles. The coexistence of nanoparticles and twins is observed in Sb containing alloys, with a negative correlation in the corresponding number density, owing to the competitive stacking of precursors and individual atoms at the solid–liquid interface. Large size particles around 70 nm with a uniform distribution of Sr atoms are formed in Al–10Si–0.35Sb–0.015Sr (wt. %) alloys, due to the precursor aggregation and homogeneous nucleation in the droplets that nucleation are depressed. A model for the formation of nanoparticles and their interaction with twins is proposed.

## 1. Introduction

Al–Si alloys have been widely used in machinery, automobiles, and aircraft, due to high specific strength, liquidity, and price ratio. With the progress of technology, studies on microstructure evolution to further improve mechanical performance have drawn more and more attention, such as decreasing the secondary dendrite arm spacing [1], eliminating solidification defects [2], and modification [3,4,5,6,7]. As is well-known, the mechanical properties of Al–Si alloys are mainly determined by the morphology and size of Si phase. Therefore, understanding and controlling the growth process of eutectic silicon is the key to improve the performance of Al–Si alloys, of which a prerequisite is to elucidate the evolution of microstructures and their interactions, such as twinning, stacking faults, and nanoparticles.

As is well-known, the interaction between twins and precipitates is not only essential for the in-depth understanding of the formation and evolution of twins during solidification, but it is also important for the strength increase during deformation [8,9]. Adversely, the interaction of twins and nanoparticles has not been well interpreted yet, due to the research difficulty in their simultaneous introduction.

It had been well-established that the twinning of eutectic Si could be easily obtained in inoculated Al–Si alloys [10,11], when trace levels of Sr or Na are added. Much attention had been devoted [12,13,14], from a scientific point of view, to the impurity induced twin (IIT) [15] and poisoning of twin plane re-entrant edge (TPRE) [16,17] mechanism. Recently, Al–Sr-rich [11,18,19] nanoparticles were observed at the intersection of Si twins, which made the eutectic Si in modified Al–Si alloys a good candidate for the interaction investigation of nanoparticles and twins. It should be mentioned that Al–rich nanoparticles coherent with the Si matrix were observed [20] to suppress the twinning of Si [21,22], which may induce the change in the nucleation path of eutectic Si. The interaction between nanoparticles and twins appeared to be controversial, which restricted the application and development of complex modifications of Al–Si alloys. Both Sb and Sr additives had been reported to induce the formation of nanoparticles in eutectic Si [18,22], however, the poisoning effect between Sr and Sb had not been clarified [23]. In order to study the evolution of eutectic Si and interaction of particles with twins, the cooling curves and microstructures of eutectic Si in the Al–10Si base and Al–10Si–0.015Sr (throughout, wt. % is used if not specified), Al–10Si–0.35Sb, and Al–10Si–0.35Sb–0.015Sr alloys have been characterized. Besides, the atomic reconstruction and liquid atomic structure were studied to illustrate the atomic mechanism during the solidification process of the Al–10Si–0.35Sb–0.015Sr alloy.

## 2. Materials and Methods

Industrially pure Al and Si were utilized to prepare the Al–10Si base alloy. The nominal Al–10Si–0.015Sr, Al–10Si–0.35Sb and Al–10Si–0.35Sb–0.015Sr alloys were manufactured with controlled additions of Al–10Sr and Al–9Sb master alloys, with a holding time of 45 min at 730 °C. Cylindrical samples for microstructure analysis were fabricated by pouring the melts into a cylindrical graphite mold (wall thickness: 10 mm; inner–diameter: 20 mm; height: 75 mm) at ambient temperature [22]. The accurate concentrations of the alloys through photoelectric direct reading spectrometry were presented in Table 1.

Ion-beam milled samples for TEM observation were obtained with a Precision Ion Polishing System (Gatan 691, Gatan Inc., Hangzhou, China). The Sr-containing samples were examined with TEM (Tecnai G2 F20, FEI, Shanghai, China), while the Al–10Si–0.35Sb alloy was observed with a JEM 2100F TEM (JEOL, Shanghai, China). The samples for Laser Pulsed APT analysis [24,25] were milled with a Focused Ion Beam (FIB–SEM, FEI Helios 600i, FEI, Shanghai, China) and examined with the LEAP™ 4000XHR (CAMECA, CAMECA, Shanghai, China) at a pulse rate of 200 kHz. The specimen temperature was approximately 50 K and the target evaporation rate was 1% under a vacuum of 10^−9^ Pa. The Integrated Visualization and Analysis Software (IVAS 3.6.4, CAMECA, Shanghai, China) was used for 3D reconstructions.

Utilizing the Vienna ab initio simulation package 11 (VASP, Vienna University, Jinan, China), ab initio molecular dynamics simulation (AIMD) was carried out with implementing the projector augmented wave method to analyze the partial coordination relationship. Taking accounting into the low Sb and Sr concentration in modified Al–Si alloys, the simulations were performed in melts with high modifiers concentration [26]. Then, dynamical simulations of liquid Si–18.2Sb–2.6Sr (Si–5at.% Sb–1at.% Sr, in atomic percentage) alloy were carried out to analyzed the atomic structure of Sb and Sr atoms in Al–10Si–0.35Sb–0.015Sr melts. The simulations were carried out in the canonical ensemble (NVT) through a Nosé thermostat to control temperature, in order to get precise local atomic structure of Sr and Sb atoms. A cubic cell containing 300 atoms was utilized with periodic boundary conditions, which was adjusted to achieve an average external pressure around zero. The alloys were equilibrated for 3 ps. Then, configurations were simulated for 6 ps with the time step of 3 fs to produce structural function at 1727 °C. Then, a thousand of configurations were used to calculate the atomic coordination [27].

## 3. Results

### 3.1. Cooling Curves

**Figure 1:**
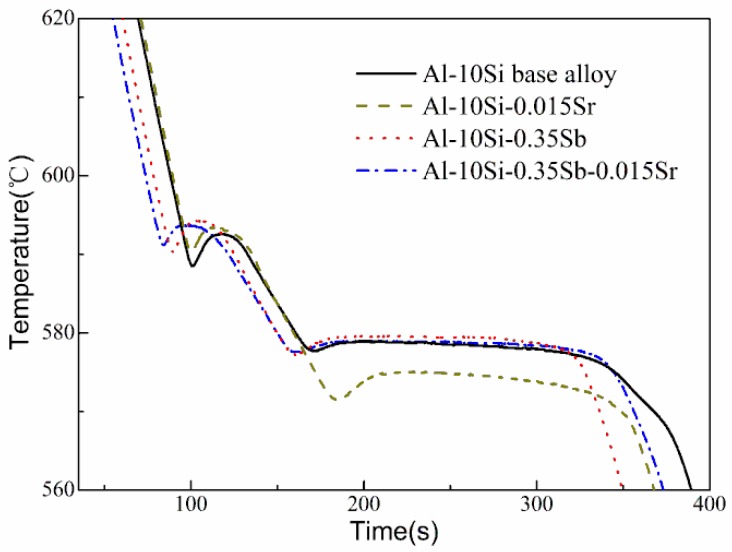
Cooling curves of Al–Si base and modified alloys.

The measured cooling curves of Al–Si based and modified melts, and the corresponding characteristic temperature, are presented in Figure 1 and Table 2, respectively. The nucleation temperature *T*_N_ is defined as the first remarkable change in the derivative of the cooling curve. The minimum temperature *T*_M_ and the growth temperature *T*_G_, are defined as the minimum prior to recalescence and the maximum temperature reached after recalescence, respectively. The eutectic nucleation temperature (*T*_N,eutectic_) is 578.4 °C for the base alloy, which is very close to the previous results [28]. With Sr additions, *T*_N,eutectic_ decreases to 572.6 °C owing to the depressed nucleation of eutectic Si. It should be noticed that the combined addition of Sb and Sr, as well as the exclusive addition of Sb, slightly depresses the nucleation of eutectic Si. It is indicated that the role of Sr is positioned by the Sb element in the Al–10Si–0.35Sb–0.015Sr alloy. An average cooling rate of about 1.4 °C/s was obtained from the cooling curves.

### 3.2. Eutectic Si Morphology

**Figure 2:**
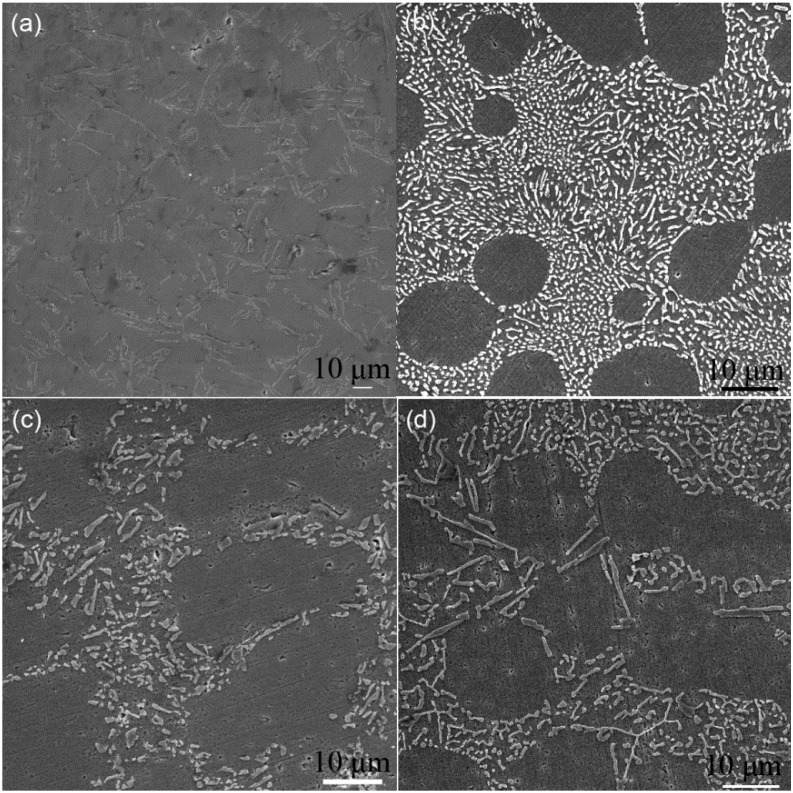
Microstructure of Al-Si base and modified alloys. (**a**) Base alloy. (**b**) Al–10Si–0.015Sr. (**c**) Al–10Si–0.35Sb. (**d**) Al–10Si–0.35Sb–0.015Sr.

The morphologies of eutectic Si (in light color) in the base alloy and those modified characterized by SEM are depicted in Figure 2a–d, since the interaction between nanoparticles and defects would influence the nucleation and growth of eutectic Si. Coarse Si plates are observed in the base alloy. The fiber-like and lamellar morphology are obtained in Sr and Sb exclusively modified alloys, respectively. When both Sr and Sb are added with a hold time of 45 min, both short rod-like and lamellar Si are observed in the morphology.

### 3.3. Cystal Defects by TEM

**Figure 3:**
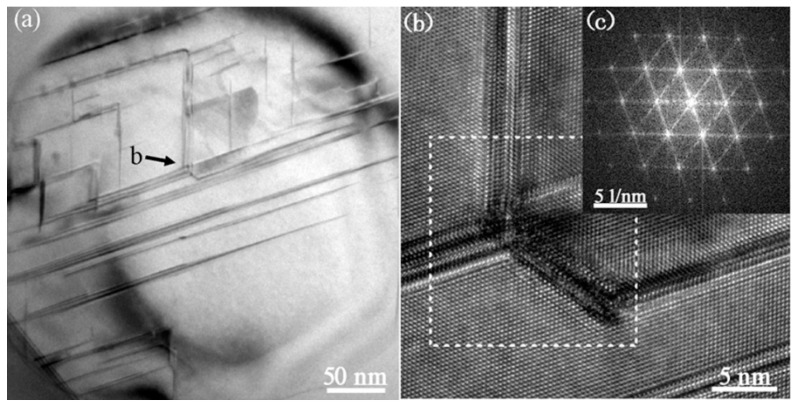
TEM image of eutectic Si in Al–10Si–0.015Sr alloy, (**a**) TEM bright–field image, (**b**) atomic lattice image at the intersection of twins as denoted by b arrow in (**a**), and (**c**) FFT image of denoted zone in dashed box.

Figure 3a exhibits the multiple twins within the eutectic Si of the Al–10Si–0.015Sr alloy. No visible particles are obtained at the intersection of multiple twins from the high-resolution lattice images presented in Figure 3b. The corresponding diffraction spots are indexed to Si atoms in Figure 3c. It appears that it is a single Sr atom rather than the Sr-rich nanoparticles that induced the multiple twins of eutectic Si.

### 3.4. Interaction of Nanoparticles with Twins

**Figure 4:**
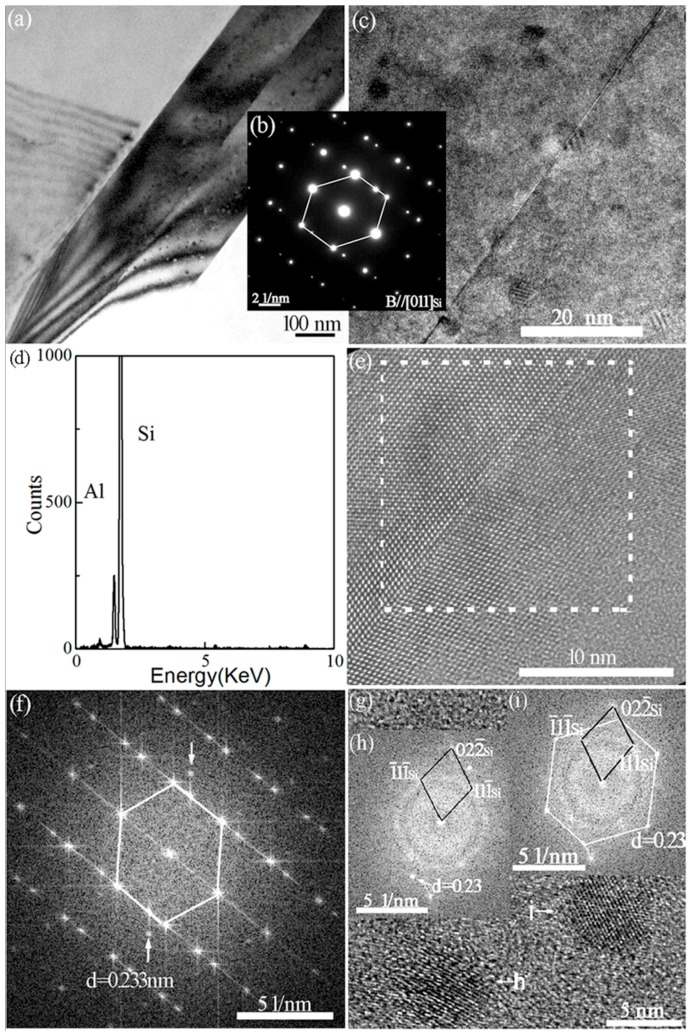
Microstructure within eutectic Si of Al–10Si–0.35Sb alloys. (**a**) TEM dark–field image, (**b**) SAED patterns, (**c**) high number density nanoparticles, (**d**) EDS spectrum, (**e**) HRTEM image of particle penetrated by twin boundary, (**f**) FFT of lattice image marked with dotted box in (**e**), (**g**) HRTEM lattice image of nanoparticles, (**h**) FFT of lattice image denoted as “h” in (**g**), and (**i**) FFT of lattice image denoted as “i” in (**g**).

Quite different from Figure 3, an eutectic Si grain with straight grain boundary and high number density nanoparticles was observed in the Al–10Si–0.35Sb alloy, as presented in Figure 4a,b. As magnified along [011]_Si_ axis and presented in Figure 4c, many nanoparticles with stacking faults are visible to be embedded in eutectic Si twins, which indicates the structural rearrangement of particles during the propagation of twins. By contrast, it is quite interesting that with similar radii, several nanoparticles are just penetrated by the twin boundary. As presented in Figure 4d, the existence of Al in Si twins is verified by the energy dispersive spectrometer (EDS) results. Following, the existence of Al atoms of the {111}_Al_ plane group in the nanoparticles penetrated by the twin boundary is revealed by the high resolution TEM lattice image in Figure 4e and spots with a interplanar distance of 0.23 nm in the corresponding FFT patterns in Figure 4f. For the nanoparticles far from the twin boundary, the spots corresponding to the {111}_Al_ plane group in the FFT patterns that are presented in Figure 4h,i, indicate that the corresponding atomic arrangements are completely consistent with the atomic arrangements of the nanoparticles penetrated by the twin boundary.

**Figure 5:**
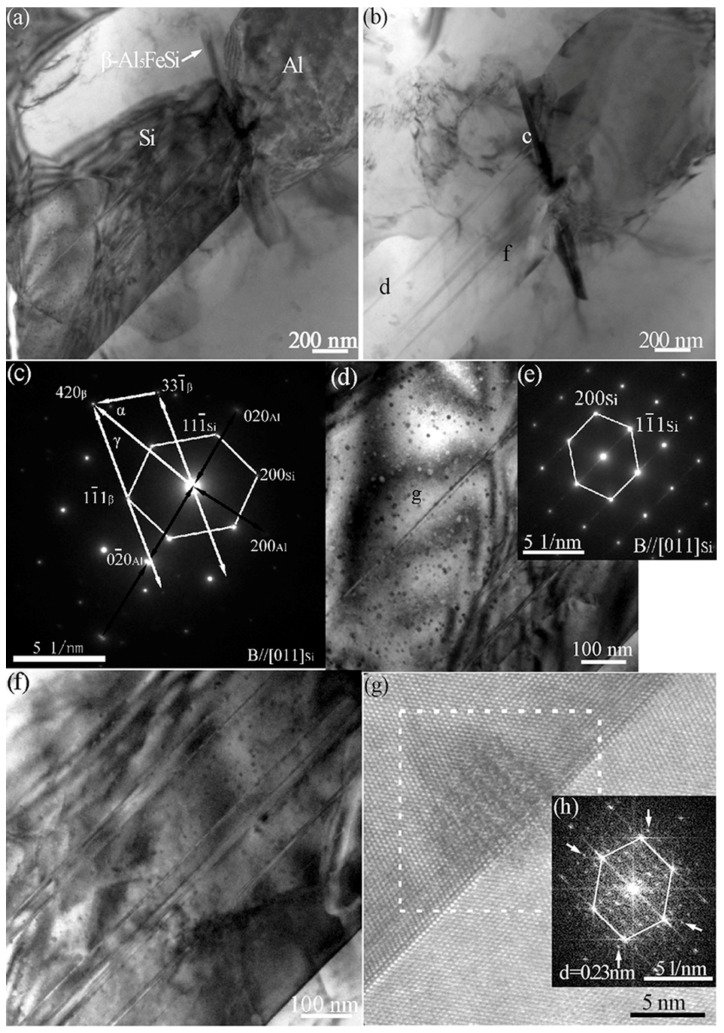
Microstructure of eutectic Si in Al–10Si–0.35Sb–0.015Sr alloy. (**a**) TEM dark–field image, (**b**) bright–field image, (**c**) SAED patterns of the zone marked with “c” in (**b**), subscript β denotes β–Al5FeSi compound, α and γ denote included angle between spots, (**d**) nanoparticles far from twin boundary, as denoted by “d” in (**b**), (**e**) SAED patterns for zone ”d” in (**b**), (**f**) nanoparticles adjacent to twin boundary, as denoted by “f” in (**b**), (**g**) nanoparticle penetrated by stacking faults, and (**h**) FFT of lattice image marked with dotted box.

The TEM bright and dark field images of the Al–10Si–0.35Sb–0.015Sr alloy are presented in Figure 5a,b. Apparently, a plate-like β–Al_5_FeSi phase is located at the interface of eutectic Si and Al grains. As presented in Figure 5c, an orientation relationship of [011]_Si_ of 11.8° from the [$\bar{1}$23]_β-Al5FeSi_ indicates that the β–Al_5_FeSi phase is not the heterogeneous nucleus of eutectic Si. Consequently, the Fe-rich particles are pushed out of the solid–liquid interface during solidification. As for the eutectic Si grain, one important characteristic is the increased density of Si twins compared to that in Figure 4a, owing to the addition of Sr into the Al–10Si–0.35Sb alloy. Another characteristic is the coexistence of nanoparticles and twins, with a negative correlation in the corresponding number density, as observed in Figure 5d,f. The lower the twin boundary spacing is, the higher number density of the nanoparticles is. Similarly to that in Figure 4e, an Al-rich nanoparticle is observed to be penetrated by the stacking faults, as presented in Figure 5g. In the corresponding FFT image, several spots corresponding to the {111}_Al_ plane group exist, as presented in Figure 5h.

### 3.5. Ab initio Molecular Dynamics Simulation

**Figure 6:**
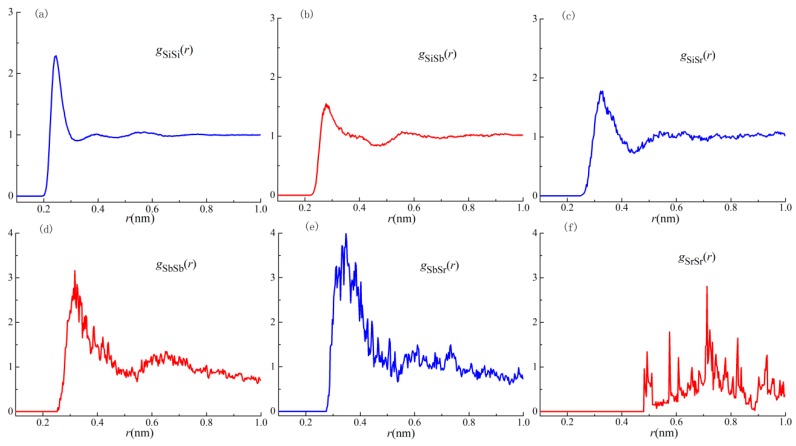
PDFs in the Si–18.2Sb–2.6Sr melts.

In order to assess the atomic structure in the melts, liquid Si–18.2Sb–2.6Sr alloy is investigated by AIMD at 1723 °C. All the partial pair distribution functions (PDFs) and partial coordination numbers (PCNs) are exhibited in Figure 6 and Table 3, respectively. Obviously, the maximum of *g*_SbSb_(*r*) and *g*_SbSr_(*r*) curves are over 3, which are far higher than those in other curves. These mean the strong affinity of Sb–Sb and Sb–Sr pairs, which may be responsible for Sb–Sb segregation and poisoning effect during modification, respectively. The low maximum of the *g*_SiSr_(*r*) curve is no more than 2, which demonstrates the weak interaction between Si–Sr pairs. Moreover, the chemical short-range order (CSRO) parameter for Sb–Sr pair α_SbSb_ was −1.410 according to α_AB_ = 1–*N*_AB_/(*c*_B_*N*_AA_ + *c*_B_*N*_AB_+ *c*_B_*N*_AC_), where *c*_B_ was the concentration of B component, *N*_AA_, *N*_AB_, and *N*_AB_ are PCNs of *g*_AA_(*r*), *g*_AB_ (*r*), and *g*_AC_(*r*) curves [29], respectively. This value reveals the strong affinity between the Sb–Sr pair. It should be noticed that both CSRO_Si-Sb_ and CSRO_Si-Sr_ show a large negative value, which implies that the nucleation of eutectic Si would be hindered by the preferential arrangement of modifier atoms around Si atoms in the exclusively modified melts.

### 3.6. Atom Probe Tomography

**Figure 7:**
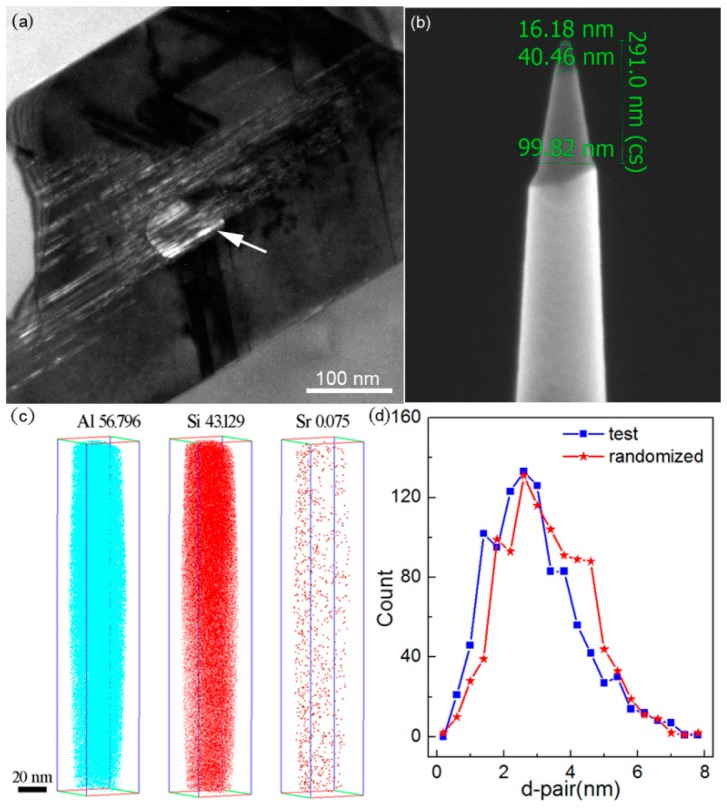
APT results of eutectic Si in Al–10Si–0.35Sb–0.015Sr alloys. (**a**) Large size nanoparticle, (**b**) SEM image of tested tip, (**c**) distribution of atoms in eutectic Si, and (**d**) nearest neighbor distribution of Sr atoms in eutectic Si.

With a diameter around 70 nm, several large particles are observed in eutectic Si of Al–10Si–0.35Sb–0.015Sr alloys, as shown in Figure 7a. To probe into the behavior of defects, the distribution and concentration of elements within such particles are characterized by three dimension atom probe tomography (APT). The present reconstructed volume is up to 37.4 × 37.4 × 220.7 nm^3^, with a uniform distribution of Al, Si, and Sr atoms as presented in Figure 7c. The mass fraction of Al in eutectic Si is up to 56.796%, which is significantly higher than the solid solution limit in the equilibrium solidified Al–Si alloys [12,19]. Typically, the nearest neighbor distribution indicates that the Sr atoms are randomized in eutectic Si, as presented in Figure 7d. However, no twin or particles is observed in Figure 7c. Considering that the Sr concentration, which is approximately 0.075%, is sufficiently high to modify the eutectic Si [30]. Then, the poisoning effects due to multiple modifications could be excluded. Nevertheless, the formation and existence of such large particles are amazing.

## 4. Discussion

In terms of the morphology of eutectic Si, lamellar and short-rod like Si are obtained in Al–10Si–0.35Sb–0.015Sr alloys. However, the characteristic temperatures in the solidification curves indicate the nucleation of eutectic Si is slightly depressed with an undercooling of 0.1 °C, as well as that in the Al–10Si–0.35Sb alloy. The low undercooling could be attributed to the long holding time and the poisoning effect between Sb and Sr additives, which indicates the morphology is not determined by the nucleation process. Since the Sb–Sr chemical short range order is revealed by AIMD results in Figure 6, the corresponding compound is concluded to be Sb_10_Sr_11_ with a tetragonal crystalline structure. However, the increased twin density is obtained owing to the addition of Sr in the Al–10Si–0.35Sb–0.015Sr alloy. Then, the refined Si morphology indicates that Sr takes a more important role than being poisoned during the growth of eutectic Si. As shown in Figure 5f, the lower the twin boundary spacing is, the higher number density of the nanoparticles is. Thus, the formation of nanoparticles and their competitive growth with twins process during the eutectic growth, which would take an important role in the morphological transition. These facts are in good agreement with the “restricted growth theory” for modification.

Observed from the TEM image, no particles could be found at the intersection of Si twins in Sr exclusively modified alloys. It indicates that single Sr atom is absorbed at the front of solid–liquid interface, which induces the formation of multiple twins and hinders the growth of eutectic Si. However, the coexistence of twins and nanoparticles is observed in Sb exclusively modified alloys. Similar coexistence is also observed in the Al–10Si–0.35Sb–0.015Sr alloy. As denoted by the FFT image in Figure 4f,h and Figure 5h, all the nanoparticles have similar size, atomic arrangement, and composition in Sb-containing alloys. These facts mean such nanoparticles are formed following the same mechanism. Compare Figure 4 with Figure 5, the penetration of Al-rich particles by the twin boundary or stacking faults is a common phenomenon, which is independent of the Sr concentration. Actually, the heterogeneous nuclei of eutectic Si are never penetrated by twin boundaries, such as TiC_0.5_N_0.5_ nanoparticles [31] and AlP particles [32], since they are solid phase in front of the solid–liquid interface. This in turn verifies that Al-rich nanoparticles are not the nuclei, but are formed during the eutectic growth and Si twinning. This conclusion is consistent with the fact that the nanoparticles far from the twin boundary have higher Al concentration, as depicted in Figure 4h,i. The number density and its interaction with twins are quite similar to that of intermetallic compound precipitates with stack faults during the deformation of Mg alloys [9], which restricts the motion of crystal defects.

Usually, the in-homogeneity of the microstructure and composition is a universal phenomenon in solidification. As exhibited in Figure 7a, a large size particle with a diameter approximately 70 nm was observed. It is very interesting that no defect or composition fluctuation is observed from the APT results. The only one reasonable explanation is the frozen issue owing to the rapid solidification. However, the bulk alloys always induced heterogeneous nucleation rather than rapid solidification. Taking account of the small radius of curvature, a high undercooling could be easily achieved in such little droplets, especially with the existence of Sr atoms. Then, once the homogeneous nucleation occurs, the homogeneous microstructure and composition would be obtained. Nevertheless, the solidification process should be deeply discussed further.

**Figure 8:**
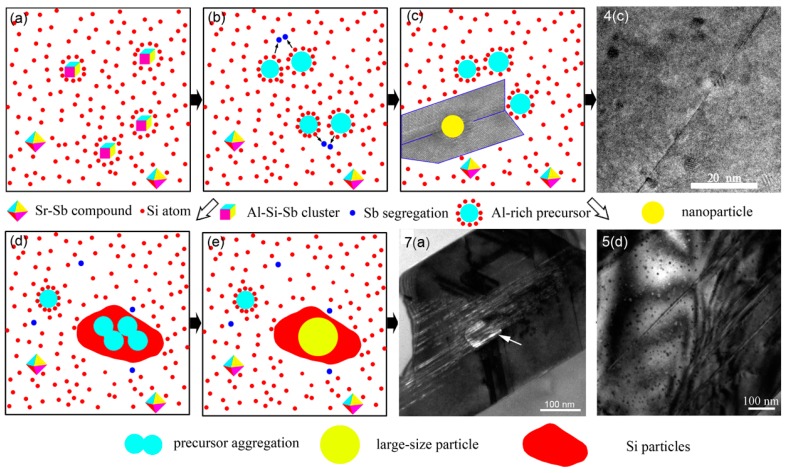
Schematic diagrams of the formation of high number density and large size nanoparticles in the Al–10Si–0.35Sb–0.015Sr alloy. Al atoms are omitted for clarity. (**a**) FCC-like Sb-Sr and Al-Si-Sb clusters in the melts, (**b**) Sb segregation and the formation of Al-rich precursors (**c**) the stacking of the whole precursors at the solid–liquid interface. (**d**) precursors aggregation within Si particle, and (**e**) large size nanoparticles formed with Si particles.

Generally, the modification elements promote the formation of clusters in the melt, such as Al–Si–Sr [15], Al–Fe–Si [21], and Al–Si–Sb [22] clusters, no matter whether the corresponding compounds are formed in the as-solidified issue or not [33,34]. The formation of a thin Al–Si–Sr layer was proposed to be formed at the front of solid–liquid interface of eutectic Si in Sr-modified Al–7Si alloys. All these facts indicate that atomic clusters containing modification elements in the Al–Si melts, as shown in Figure 8a. As depicted in Figure 6e, the maximum in the PDF curves is over 3, which indicates the strong attractive interaction between Sb–Sr pairs. The formation of intermetallic Sb_10_Sr_11_ compounds was reported in Si-rich alloys, with a tetragonal crystalline structure. Then, it is reasonable to conclude that octahedral Sb–Sr clusters exist in the melts as shown in Figure 8, since only five kinds of regular polyhedrons exist in the metallic melts. Verified by Figure 7c, for Al–Si–Sb clusters, the Al-Si-rich precursor would be formed since Sb–Sb segregation moves out as shown in Figure 8b. During the growth of eutectic Si twins, individual Si atoms or the whole clusters would be stacked at the solid–liquid interface. Especially, the whole precursors are easy to be located at the twin plane re-entrant edges [21] and rapidly entrapped by the Si twins, as shown in Figure 8c. Then, these precursors are penetrated by twins as the liquid–solid transition and atomic rearrangement process. Then, the microstructure in Figure 4c is obtained in Al–10Si–0.35Sb alloy. However, the rearranged atoms would not rigidly follow that of Si twins and the propagation of twin would be hindered when plenty of precursors are attached in Al–10Si–0.35Sb–0.015Sr alloy, as shown in Figure 5d,f.

During the eutectic solidification of Al–10Si–0.35Sb–0.015Sr alloy, Al-Si-rich clusters and precursors would be co-aggregated owing to the surface tension, very like that in liquid phase separation [35]. Once the heterogeneous nucleation of Si particles occurs, the co-aggregating precursors would impinge with Si particles and rapidly attached at the solid–liquid interface. As demonstrated by the distribution of elements in Figure 7c, Sr atoms evenly survive in the Al-rich particles. Then, the nucleation of co-aggregating precursors would be hindered by the existence of Sr atoms, even entrapped by crystalline Si, which would also restrict the growth of eutectic Si and help to modify the eutectic Si morphology. This means such precursors have enough time to aggregate and grow up as shown in Figure 8d. Taking account of the small radius of such liquid precursors, the critical undercooling is very large. However, as precursors aggregate, the critical undercooling rapidly decreases, which may induce the homogeneous nucleation and rapid liquid–solid transition and produce the large particles without any defects, as depicted in Figure 8e. The formation of nanoparticles with a radius beyond 40 nm [20] and length beyond 220 nm [19] has been reported. Thus, the atoms in the large particles are frozen and no concentration fluctuation is observed, as shown in Figure 7a,c. The present APT reconstruction volume just locates inside the large size Al–rich nanoparticles with a length exceeding two-hundred nanometers, such as in the particle of Figure 7a.

The formation of high number density and large size nanoparticles could be well interpreted by the present model, which is owing to the rapid staking and co-aggregation of precursors, respectively. The competition of local atomic rearrangement, which would induce the formation of stacking faults inside the nanoparticles to release the free energy during eutectic Si growth, leads to the negative number density between nanoparticles and twins in Al–10Si–0.35Sb–0.015Sr alloy. The difference in the morphologies of Al–10Si–0.35Sb and Al–10Si–0.35Sb–0.015Sr alloy is attributed to the survived Sr atoms in the melts, which restricts the nucleation of aggregated droplets and induces their homogeneous nucleation inside eutectic Si. Owing to the rapid solidification of aggregated droplets, the large size particles with uniform atomic distribution and without crystalline defects are formed, which suppress the propagation of the twins. The present results seem to be quite different from the points of view that Sr-rich particles induce the formation of multiple twins. More research should be done to clarify the influence of as-formed primary and eutectic Al on the growth of eutectic Si and their inner atomic mechanisms in the future.

## Figures and Tables

**Table 1:** Chemical composition of samples (wt. %).

Alloys	Al	Si	Fe	Mn	Ni	Ti	Na	P	Sb	Sr
Al–10Si	89.844	10.010	0.141	0.001	0.001	0.001	0.001	0.001	0	0
Al–10Si–0.015Sr	90.133	9.720	0.125	0.001	0.002	0.002	0.001	0.001	0	0.015
Al–10Si–0.35Sb–0.015Sr	89.552	9.940	0.140	0.001	0.003	0.005	0.001	0.001	0.344	0.013
Al–10Si–0.35Sb	89.409	10.100	0.148	0.001	0.003	0.006	0.001	0.001	0.331	0

**Table 2:** Characteristic temperature in the cooling curves.

	T_N,α-Al_ (°C)	T_M,α-Al_ (°C)	T_N,E_ (°C)	T_M,E_ (°C)	T_G,E_ (°C)
Al–10Si	589.3	588.6	578.4	577.7	578.8
Al–10Si–0.015Sr	591.1	590.4	572.6	571.5	575.1
Al–10Si–0.35Sb	591.0	590.4	578.3	577.2	579.6
Al–10Si–0.35Sb–0.015Sr	592.0	591.2	578.3	577.6	578.9

**Table 3:** Calculated partial coordination number (Ni-j) and chemical short-range order (CSRO).

*Ni*-*j*	*j* = Si	*j* = Sb	*j* = Sr	CSRO_i-Si_	CSRO_i-Sb_	CSRO_i-Sr_
*i* = Si	6.668	0.887	0.130	0.077	−1.308	−0.700
*i* = Sb	16.673	1.573	0.452	0.051	−0.682	−1.410
*i* = Sr	12.250	2.258	0.023	0.103	−2.108	0.840
